# Adjuvanted Fusion Protein Vaccine Induces Durable Immunity to *Onchocerca volvulus* in Mice and Non-Human Primates

**DOI:** 10.3390/vaccines11071212

**Published:** 2023-07-06

**Authors:** Nathan M. Ryan, Jessica A. Hess, Erica J. Robertson, Nancy Tricoche, Cheri Turner, Jenn Davis, Nikolai Petrovsky, Melissa Ferguson, William J. Rinaldi, Valerie M. Wong, Ayako Shimada, Bin Zhan, Maria Elena Bottazzi, Benjamin L. Makepeace, Sean A. Gray, Darrick Carter, Sara Lustigman, David Abraham

**Affiliations:** 1 Department of Microbiology and Immunology, Sidney Kimmel Medical College, Thomas Jefferson University, Philadelphia, PA 19107, USA; 2 Laboratory of Molecular Parasitology, Lindsey F. Kimball Research Institute, New York Blood Center, New York, NY 10065, USA; 3 PAI Life Sciences Inc., Seattle, WA 98102, USA; 4 Vaxine Pty Ltd., Bedford Park, Adelaide, SA 5042, Australia; 5 Alpha Genesis Inc., Yemassee, SC 29945, USA; 6 IDEXX BioAnalytics, West Sacramento, CA 95605, USA; 7 Division of Biostatistics, Department of Pharmacology and Experimental Therapeutics, Sidney Kimmel Medical College, Thomas Jefferson University, Philadelphia, PA 19107, USA; 8 Texas Children’s Hospital Center for Vaccine Development, Department of Pediatrics, National School of Tropical Medicine, Baylor College of Medicine, Houston, TX 77030, USA; 9 Institute of Infection, Veterinary & Ecological Sciences, University of Liverpool, Liverpool L3 5RF, UK

**Keywords:** *Onchocerca volvulus*, vaccine, durability, adjuvant, mice, non-human primates, Advax, fusion protein, passive immunization, river blindness

## Abstract

Onchocerciasis remains a debilitating neglected tropical disease. Due to the many challenges of current control methods, an effective vaccine against the causative agent *Onchocerca volvulus* is urgently needed. Mice and cynomolgus macaque non-human primates (NHPs) were immunized with a vaccine consisting of a fusion of two *O. volvulus* protein antigens, *Ov*-103 and *Ov*-RAL-2 (*Ov*-FUS-1), and three different adjuvants: Advax-CpG, alum, and AlT4. All vaccine formulations induced high antigen-specific IgG titers in both mice and NHPs. Challenging mice with *O. volvulus* L3 contained within subcutaneous diffusion chambers demonstrated that *Ov*-FUS-1/Advax-CpG-immunized animals developed protective immunity, durable for at least 11 weeks. Passive transfer of sera, collected at several time points, from both mice and NHPs immunized with *Ov*-FUS-1/Advax-CpG transferred protection to naïve mice. These results demonstrate that *Ov*-FUS-1 with the adjuvant Advax-CpG induces durable protective immunity against *O. volvulus* in mice and NHPs that is mediated by vaccine-induced humoral factors.

## 1. Introduction

*Onchocerca volvulus* is the causative agent of onchocerciasis, a debilitating neglected tropical disease primarily endemic in sub-Saharan Africa, with an estimated 21 million people infected. Onchocerciasis is characterized by severe ocular, lymphatic, and dermal pathology. These disease manifestations contribute to the estimated 1.4 million disability-adjusted life years lost globally in 2020 [1]. The primary control method for preventing *O. volvulus* disease and transmission is mass drug administration (MDA) of ivermectin. As a microfilaricide, ivermectin is not effective at killing adult parasites, and it has been estimated that elimination would require biannual administration for 15–35 years [2]. Other challenges with ivermectin-based MDA include non-compliance [3], emerging resistance [4], lethal adverse effects with *Loa loa* co-infection [5], and lack of approval for use in children under five years of age [6]. Collectively, these obstacles explain why only an estimated 31% reduction in *O. volvulus* prevalence occurred in Africa between 1990 and 2013. Therefore, elimination will likely require the addition of new therapies and, most importantly, a prophylactic vaccine [7,8,9].

Studies of vaccine candidates and immune responses to *O. volvulus* in small animal hosts have been conducted using challenge infections contained within subcutaneously implanted diffusion chambers. Diffusion chambers consist of Lucite rings covered with 5.0 µm pore-size membranes, which allow host effector cells and soluble factors to diffuse freely into and out of the parasite microenvironment while preventing loss of challenge larvae by dissemination [10]. Using diffusion chambers implanted in mice, a vaccine against *O. volvulus* has been identified that consists of two recombinant *O. volvulus* antigens, *Ov*-103 and *Ov*-RAL-2, which are expressed by infective third-stage larvae (L3). These two antigens were selected from a pool of 44 recombinant *O. volvulus* antigens, 15 of which were observed to induce protective immunity. Further selection of seven *O. volvulus* antigens was based on whether candidates were nematode- or parasite-specific, could be localized to *O. volvulus* larvae, were recognized by immune serum from putatively immune humans and immunized animals, and those with homologs found to be protective in other parasitic infections [11]. *Ov*-103 and *Ov*-RAL-2 were selected based on their ability to induce the most consistent and highest level of protection in the mouse diffusion chamber model [12]. *Ov*-103 is expressed by microfilariae, L3 and adult stages in the hypodermis, and the basal layer of the cuticle [13]. *Ov*-RAL-2 is expressed in the hypodermis of L3 and adult-stage *O. volvulus* [11]. Furthermore, the development of *Ov*-RAL-2-specific antibodies in infected individuals has been associated with a reduced chance of developing ocular opacities [14]. When formulated in combination with either Advax-CpG or alum as the adjuvant, *Ov*-103 and *Ov*-RAL-2 consistently induced protective immunity against larval challenge in mice [12,15,16]. Vaccination of naïve calves with *Ov*-103 or *Ov*-RAL-2 formulated with Montanide significantly reduced the rate of acquisition of *O. ochengi* nodules and microfilaridermia over 24 months of exposure to natural infections [17]. Furthermore, both antigens were detected by antibodies from putatively immune humans and those who developed concomitant immunity with age, thus demonstrating their relevance in human protective immunity [18].

The present study investigates the immunogenicity and efficacy of a vaccine consisting of a bivalent fusion of the two *O. volvulus* antigens, *Ov*-103 and *Ov*-RAL-2 (*Ov*-FUS-1), in mice and NHPs. The cost of a clinical vaccine against *O. volvulus* will be important and there is an economic advantage of a fusion antigen, as only a single cGMP protein needs to be manufactured, purified, and shown to be stable and safe. Using a single fusion protein will also ease the challenges of producing and distributing the vaccine in clinical trials and, eventually, large-scale vaccination campaigns in endemic countries [19,20,21]. Comparative analysis of different antigen constructs demonstrated that fusion antigens induced equivalent protection to the individual antigens [22,23]. A multivalent fusion protein, developed for a vaccine against the filarial worm *Brugia malayi* (r*Bm*HAXT), was shown to induce significant protective immunity in gerbils, mice, and Rhesus macaques [24,25,26,27]. *B. malayi* orthologues of *Ov*-103 and *Ov*-RAL-2 prepared as a fusion formulated with alum revealed equivalent efficacy in immunized gerbils compared to the individual antigens [23].

Adjuvants can influence the durability of immune responses [28,29]; therefore, we evaluated the efficacy of *Ov*-FUS-1 when formulated with three different adjuvants: (1) Advax-CpG is a combination adjuvant containing delta inulin and CpG oligodeoxynucleotide (CpG) [30] and, when administered with helminth antigens, has been shown to induce a mixed helper T cell type 1 (Th1)/helper T cell type 2 (Th2) response [15,16]. Delta inulin is an isoform of the plant-derived polysaccharide, inulin, which has been shown to be safe in human clinical trials [31,32,33,34]. Delta inulin-based adjuvants activate the alternative complement pathway, enhance the chemotaxis of mononuclear cells to injection sites, and upregulate costimulatory molecules on these cells, thereby improving antigen presentation and lymphocyte activation [30,31]. Delta inulin has been incorporated into multiple vaccines, where it was shown to enhance vaccine immunogenicity and provide dose-sparing effects [30,32,35,36]. The CpG component of Advax-CpG mimics the CpG motifs common in bacterial DNA and interacts with the intracellular pattern recognition receptor, TLR9, to stimulate a Th1-biased response [37,38]. (2) Alum is composed of aluminum hydroxide and stimulates a Th2-polarized response. It has been safely used in human adults and children for decades [39,40]. Alum has been observed to recruit macrophages, dendritic cells, neutrophils, and eosinophils to the injection site, where the particulate adjuvant aids in antigen delivery to and uptake by antigen-presenting cells [39,41]. (3) AlT4 is a co-formulation of alum with a TLR4 agonist and induces a mixed Th1/Th2 response through the activation of the Nf-κB and MAPK pathways, resulting in enhanced expression of pro-inflammatory genes and costimulatory molecules [42]. TLR4 activation has been shown to enhance protective immunity toward *O. volvulus* larvae but is not necessary for the induction of a Th2 response [43].

The goal of the present study was to identify the best vaccine formulation that is effective against *O. volvulus* in mice and NHPs. Mice were selected for this study based on extensive previous experience with the vaccine in mice [12,15,16], and because of the availability of specific reagents for studying murine immune responses. NHPs were selected for parallel studies to validate the results in a second species more genetically similar to humans and to build a body of evidence for the clinical translation of an *Ov*-FUS-1-based vaccine. The results obtained from this study demonstrate that vaccination with *Ov*-FUS-1 and either Advax-CpG, alum, or AlT4 adjuvants is immunogenic in mice and NHPs based on the induced antigen-specific antibody responses. *Ov*-FUS-1 with either Advax-CpG or alum induced a protective immune response that resulted in a significant reduction in *O. volvulus* L3 survival in mice. In both mice and NHPs, the protective immune response induced by *Ov*-FUS-1 with Advax-CpG proved to be more durable than formulation with the other adjuvants.

## 2. Materials and Methods

### 2.1. Source of Parasites

*O. volvulus* L3 were collected from newly emerged adult *Simulium damnosum* after feeding on consenting infected donors (Protocol 320, approved by the New York Blood Center and the Medical Research Station, Kumba, Cameroon, institutional review boards). *S. damnosum* were housed in a controlled insectary and dissected after one week to collect, clean, and cryopreserve developed L3, as previously described [44].

### 2.2. Source of Mice and NHPs

Male BALB/cByJ mice, aged six to eight weeks, were acquired from The Jackson Laboratory (Bar Harbor, ME, USA). Mice were maintained in the Thomas Jefferson University Laboratory Animal Sciences Facility and housed in micro-isolator boxes in specific pathogen-free rooms under temperature, humidity, and light cycle-controlled conditions. Water and autoclaved rodent chow were provided to mice ad libitum (Philadelphia, PA, USA). Twelve male cynomolgus macaques (*Macaca fascicularis*) used in this study were housed at Alpha Genesis, Inc. (Yemassee, SC, USA). The macaques were each approximately five to eight years of age and averaged 7.05 kg in weight. The physical properties of each macaque were evaluated before inclusion in the experiment, and all were determined to be in excellent health.

### 2.3. Animal Ethics

The animal use protocol (00136) was approved by the Thomas Jefferson University Institutional Animal Care and Use Committee (IACUC). Protocols and procedures were conducted in compliance with the ethical and regulatory standards for animal experimentation set by the National Institute of Health (NIH). All animal use protocols adhered to the “Guide for the Care and Use of Laboratory Animals” published by the National Research Council, USA.

All *M. fascicularis* animal use protocol methods were approved by an IACUC committee prior to initiation. Protocols and procedures were conducted in accordance with the US National Research Council’s Guide for the Care and Use of Laboratory Animals, the US Public Health Service’s Policy on Humane Care and Use of Laboratory Animals, and the Guide for the Care and Use of Laboratory Animals. All experimental procedures were performed by certified veterinarians and technicians in accordance with the set guidelines for animal care.

### 2.4. Production of Ov-103 and Ov-RAL-2 Antigens

Recombinant *Ov*-103 was produced as a 6× histidinyl-tagged soluble protein in the PichiaPink yeast expression system. Recombinant *Ov*-RAL-2 was expressed in the *Escherichia coli* strain BL21. Vaccine antigens were produced and purified following previously established protocols [12]. A Q anion-exchange column was used to remove endotoxin to less than 2.7 EU/mg.

### 2.5. Cloning and Cell Banking of Ov-FUS-1 Antigen

*Ov*-FUS-1 protein was designed as a fusion of the open reading frames of the proteins *Ov*-103 and *Ov*-RAL-2, separated by a 15-amino acid glycine-serine linker comprised of the sequence GGGGSGGGGSGGGGS [(G4S)3]. The gene was codon-optimized for expression in *P. pastoris* X-33, cloned into the vector pPICZ-alpha A, and transformed into *E. coli* Turbo (New England Biolabs, Ipswich, MA, USA) cells. Resulting sub-clones were confirmed by PCR amplification and Sanger sequencing, transformed into *P. pastoris* strain X-33, and selected on YPD plates containing 100 µg/mL Zeomycin. Yeast transformants were grown in Buffered Complex Glycerol Medium (BMGY) broth and induced by diluting in Buffered Methanol-Complex Medium (BMMY) (*Pichia* expression kit manual, Invitrogen, Waltham, MA, USA). Crude supernatants were analyzed using SDS-PAGE for the presence of the ~32 kDa band corresponding to the expected molecular weight (MW) of *Ov*-FUS-1. One clone exhibiting high expression of *Ov*-FUS-1, clone *Ov*-FUS-1-9H, was prioritized for large-scale production and cell banking.

### 2.6. Production of Ov-FUS-1 by Fermentation in P. pastoris

Fermentation development was pursued at the 2 L scale and a fermentation batch record was developed. An Applikon ez-Control Bioprocessor (Applikon Biotechnology Inc., Foster City, CA, USA) was used for the fermentation. The *Pichia* system was selected based on the efficient production of protein without bacterial endotoxins. Briefly, one vial of *P. pastoris* clone *Ov*-FUS-1-9H was inoculated into 200 mL of BMGY broth and grown overnight aerobically at 30 °C with 250 rpm shaking. The overnight culture was used to inoculate a 5 L fermentation vessel containing 2 L of BMGY media. The pH was monitored and maintained at pH 4.0 using 6 N HCl or 5 N NaOH as needed. Yeast cultures were bulked by feeding glycerol at 12 g/hour and monitoring cell densities by taking hourly OD_600_ readings. After five—eight hours, when yeast densities reached at least 75 OD_600_, the glycerol feed was stopped, and the dissolved oxygen was monitored and maintained at 30%. Once glycerol depletion was confirmed, the temperature was reduced to 25 °C, and expression of *Ov*-FUS-1 protein was induced by adding methanol at 7.5 g/hour/L. Induction was continued overnight for 14–20 h. Expressed *Ov*-FUS-1 was isolated from the growth medium after centrifugation at 8000× *g* for 15 min to pellet yeast. The pellets were discarded, and the clarified media was filter-sterilized through a 0.45 μm filter. Clarified media was concentrated five- to tenfold by tangential flow filtration chromatography and then buffer-exchanged into 50 mM Tris pH 8.0 using at least ten volumes of 50 mM Tris pH 8.0. The final buffer-exchanged protein bulk was filter-sterilized through 0.22 µm filters.

### 2.7. Purification of Ov-FUS-1 by Chromatography

*Ov*-FUS-1 protein was purified on an ÄKTA Pure Chromatography system (Global Life Sciences Solutions USA LLC, Marlborough, MA, USA). The *Ov*-FUS-1 protein was purified by first binding to Capto MMC (Global Life Sciences Solutions USA LLC) resin. The protein was passed over the resin at a flow rate of one–two mL/min, followed by washing with two column volumes (CV) of 50 mM Tris pH 8.0, followed by two CV of 0.1 M NaCl/50 mM Tris pH 8.0. Protein was eluted using 400 mM NaCl/50 mM Tris pH 8.0. The Capto MMC elution bulk was adjusted to 1.7 M Ammonium Sulfate/50 mM Tris/pH 8.0, and the protein was polished by passage across Capto Butyl ImpRes (Global Life Sciences Solutions USA LLC) resin. The Butyl resin was washed with at least two CV of Butyl Buffer A (1.7 M Ammonium Sulfate/50 mM Tris/pH 8.0) and two CV of 0.68 M Ammonium Sulfate. Bound *Ov*-FUS-1 was eluted with 50 mM Tris/pH 8.0. The final elution bulk was buffer-exchanged with ten volumes of 50 mM Tris pH 8.0 using a Pellicon Tangential Flow Filtration Apparatus outfitted with a 10,000 Da MW cut-off cassette. Concentration was determined by measuring absorbance at λ = 280 nm and adjusted to a final concentration of 1.5–2.0 mg/mL. Purified protein was filter-sterilized through a 0.22 µm filter, and protein was stored at −80 °C. To evaluate the purity and identity of the antigen, three 1 μg aliquots of *Ov*-FUS-1 and bovine serum albumin (BSA) standard were separated on a 4–20% Tris-glycine SDS-PAGE gel under reducing conditions and analyzed using ImageJ densitometry to confirm both the concentration and purity. Specificity was confirmed by Western blot analysis using specific murine *Ov*-FUS-1 antisera. The sera were used at 1:5000 and detected with goat anti-mouse IgG (H+L) HRP antibody at a 1:10,000 dilution (Thermo Fisher Scientific, Waltham, MA, USA).

### 2.8. Mouse Immunization and Challenge Protocol

For experiments comparing the co-administered, combination, and fusion vaccine formulations, all immunizations consisted of appropriate antigen mixtures with Advax-CpG (Vaxine Pty Ltd., Adelaide, Australia). The “co-administered” formulation consisted of two separate formulations of 25 µg of either *Ov*-103 or *Ov*-RAL-2 with 0.5 mg Advax-CpG, each brought to a total volume of 50 µL using Tris-buffered saline (TBS) (Corning, Corning, NY, USA). Both immunizations were administered intramuscularly, with each antigen formulation injected bilaterally into opposing caudal thigh muscles. The “combination” immunizations consisted of 25 µg each of *Ov*-103 and *Ov*-RAL-2 with 1 mg Advax-CpG, brought to a total volume of 100 µL in TBS. The *Ov*-FUS-1 immunization consisted of 50 µg of *Ov*-FUS-1 with 1 mg Advax-CpG, brought to a total volume of 100 µL using TBS. Both the *Ov*-FUS-1 and combination vaccines were administered as bilateral intramuscular injections of 50 µL in each caudal thigh muscle, for a total of 100 µL per mouse. Adjuvant-only controls received 1 mg Advax-CpG, brought to a total volume of 100 µL using TBS. Each mouse was immunized on Day 0 and received two additional booster injections two weeks apart. Each immunization was prepared within one hour of administration.

Diffusion chambers were constructed using 14 mm Lucite rings with 5.0 µm pore-size Durapore membranes (EMDMillipore, Billerca, MA, USA). Cryopreserved *O. volvulus* L3 were defrosted and washed, as previously reported [12,15,16], with a 1:1 solution of NCTC-135 and Isocove’s modified Dulbecco’s medium (Sigma, ST. Louis, MO, USA) and 100 U penicillin, 100 µg streptomycin (Corning), 100 µg gentamicin (EMDMillipore), and 30 µg of chloramphenicol (APP Pharmaceuticals LLC, Schaumburge, IL, USA) per mL. These antibiotics were selected based on their ability to control contaminants without influencing the survival and development of *O. volvulus* or its endosymbiont *Wolbachia* [45,46,47,48,49,50]. Each mouse was challenged with 25 *O. volvulus* L3 in a single diffusion chamber. Challenge infections were administered through subcutaneous implantation of diffusion chambers in the rear flank of each mouse. All mice received challenge infections two weeks post-final booster, and diffusion chambers were surgically removed either one, two, or three weeks later, and the contents were collected for analysis.

For experiments comparing *Ov*-FUS-1 and three different adjuvant formulations, all immunizations consisted of 50 µg *Ov*-FUS-1. Immunizations were formulated with one of the following three adjuvants: (1) 1 mg Advax-CpG. (2) 250 μg/mL alum (Rehydrogel LV 2%) (Chemtrade LLC, Berkely Heights, NJ, USA) used at a final concentration of 2.0 mg/mL aluminum. (3) AlT4 consisting of 2 mg/mL alum absorbed with 0.25 mg/mL of 3D (6-acyl)-PHAD, a synthetic TLR4 agonist modeled on bacterial monophosphoryl lipid A. All vaccine formulations were brought to a final volume of 100 µL using Tris-buffered saline (TBS) (Corning, Corning, NY, USA). Adjuvant controls received injections of equivalent amounts of adjuvant and were brought to a total volume of 100 µL using TBS. Mice were immunized and challenged as described above. Illustrations depicting the experimental timelines were generated using BioRender (BioRender, Toronto, Canada). Depending on the time point being investigated, mice received a challenge either two weeks post-final booster (early challenge time point) or ten weeks post-final booster (late challenge time point). Regardless of the time of challenge, the diffusion chambers only remained within mice for one week, followed by surgical removal and collection of the contents for analysis.

### 2.9. NHP Immunization and Challenge Protocol

Twelve cynomolgus NHPs were divided equally and randomly into four groups. The control group received 300 μL intramuscular injections of phosphate-buffered saline (PBS). The vaccine groups were vaccinated intramuscularly with *Ov*-FUS-1 formulated with one of three adjuvants—Advax-CpG, alum (Alhydrogel 2%) (Croda, East Yorkshire, UK), or AlT4. Advax-CpG was stored in a separate vial from *Ov*-FUS-1 and combined no more than one hour before each dose. Each vaccine dose consisted of 100 μg of *Ov*-FUS-1 formulated with 10 mg Advax-CpG, 2 mg/mL alum, or 25 μg AlT4, and PBS was used to bring each dose volume to 300 μL. The immunization schedule included an initial vaccination on Day 0, followed by two booster injections at four-week intervals on Days 29 and 59. Each 300 μL vaccine dose was administered as two intramuscular injections of 150 µL in each hindquarter. Approximately five weeks after the final booster on Days 91–92, NHPs were each challenged with eight diffusion chambers, five containing 25 *O. volvulus* L3 and three containing only media and no larvae. Diffusion chambers were constructed, and *O. volvulus* larvae were prepared as described above. Diffusion chambers were loaded with or without 25 L3 and implanted subcutaneously in two columns of four on the upper back of the NHPs, and the surgical incisions were sutured closed. One week later, on Days 98–99, the diffusion chambers were removed, and the contents were collected for analysis.

### 2.10. Recovery of Larvae from Mouse and NHP Diffusion Chambers

Diffusion chambers were opened by removing membranes, and contents were observed under a stereo microscope to determine the number of surviving larvae. Larvae were considered to have survived if they were found within the diffusion chamber contents and were visibly motile. The percent reduction in larval survival was calculated by [(average number of surviving larvae from control animal–average number of surviving larvae from immunized animal) ÷ average number of surviving larvae from control animal] × 100.

### 2.11. Passive Transfer of Mouse and NHP Serum into Naïve Mice

Blood was collected from each mouse at the termination of the experiment by exsanguination and pooled into three groups: (1) Naïve mice that never received any control injection, immunization, or challenge. (2) “Pre-challenge” mice that were immunized but never received challenge. (3) “Post-challenge” mice that were immunized and challenged prior to collection. Blood was left to clot at 4 °C for one hour and then centrifuged at 10,000× *g* for ten minutes at room temperature. Clots were removed with sterile forceps, and blood was centrifuged again at 100× *g* for ten minutes at room temperature to isolate serum. Serum isolated from blood samples collected from the NHPs 6 and 22 weeks post-final booster was pooled by control and adjuvant groups. All serum was filter-sterilized and stored at −80 °C until use. Thawed sera were divided into 100 µL injections using a 1 mL TB syringe with 25G × 5/8 needle (Becton Dickinson and Company, Franklin Lakes, NJ, USA). Naïve BALB/cByJ mice were challenged with 25 *O. volvulus* L3 in diffusion chambers as described above. At the time of challenge, 100 µL of pooled serum was injected subcutaneously adjacent to the diffusion chamber. Three days later, mice received another 100 µL dose of serum subcutaneously at the same location. Seven days post-challenge, diffusion chambers were surgically removed, and the contents were collected for analysis.

### 2.12. Analysis of Mouse and NHP Diffusion Chamber Cells by Microscopy

Cells were identified using microscopy for immunization experiments comparing co-administered, combination, and *Ov*-FUS-1 vaccine antigens, as well as all serum transfer studies. Dead and surviving *O. volvulus* larvae were removed from diffusion chambers at the time of recovery, and fluid containing cells was transferred to 1.5 mL Eppendorf tubes. For NHP studies, cells were recovered from the diffusion chamber fluid of three diffusion chambers containing *O. volvulus* L3 and one diffusion chamber without L3 per NHP. Cell suspensions were spun at 10,000× *g* for ten minutes at room temperature. Supernatant was removed and set aside for later analysis, and cells were suspended in equivalent volumes of PBS. Total cell counts from mice were determined using a hemocytometer (Reichert, Buffalo, NY, USA), and total cell counts from NHPs using a Countess Automated Cell Counter (Fisher Scientific). Cells were then mounted on slides using a Cytospin 3 centrifuge (Shandon, Pittsburgh, PA, USA) and stained using a Hema 3 staining kit (Thermo Fisher Scientific, Waltham, MA, USA) following the manufacturer’s protocol. Light microscopy was used to differentiate the cells based on morphology.

### 2.13. Analysis of Mouse Diffusion Chamber Cells by Flow Cytometry

For experiments comparing *Ov*-FUS-1 and three different adjuvant formulations in mice, cells were collected from diffusion chambers as described above. Erythrocytes were lysed using BD Pharm Lyse (BD Biosciences, San Jose, CA, USA) for ten minutes at room temperature. Cells were washed with FACS buffer (3% BSA (Gemini Bio-Products, West Sacramento, CA, USA), 0.5 mM ethylenediaminetetraacetic acid (Sigma-Aldrich, Burlington, MA, USA), in PBS) and filtered using a 70 µm cell strainer (Corning). Cells were centrifuged at 500× *g* for ten minutes at 4 °C, the supernatant was decanted, and pellets were resuspended in the remaining buffer. Ten µL of anti-mouse CD16/32 FC block (BioXCell, Lebanon, NH, USA) was added to each sample and incubated for ten minutes at 25 °C. Cells were stained in 100 µL total volume staining mix consisting of anti-CD49b PE-Cyanine5 (0.313 µL), anti-FceR1 PerCP-eFluor710 (0.039 µL), anti-CD335 APC (0.625 µL) (eBioscience, Dan Diego, CA, USA), anti-CD117 FITC (0.078 µL) (BioLegend, San Diego, CA, USA), anti-Ly6C Alexa Fluor 700 (0.039 µL), anti-CD11c PE-Cy7 (0.039 µL), anti-F4/80 BV421 (0.625 µL), anti-CD3e BV711 (1.250 µL), anti-CD19 BV786 (1.250 µL), anti-Ly6G PE (0.313 µL), CD11b PE-CF594 (0.156 µL), and Fixable Viability Stain 510 (0.100 µL) (BD Biosciences). Cells incubated in staining mix for 45 min at 4 °C in the dark were washed and resuspended in 200 µL of FACS buffer and 50 µL of CountBright absolute counting beads (Invitrogen, Waltham, MA, USA). Cells were analyzed on the same day using a BD LSRFortessa (BD Biosciences), and data were analyzed using FLowJo v10 (FlowJo LLC, Ashland, OR, USA). Cell populations were differentiated using a gating strategy that eliminates debris, doublets, dead cells and identified eosinophils (CD11c^−^, CD11b^+^, Ly6G^−^, Ly6C^+^, SSC^hi^), macrophages (CD11c^−^, CD11b^+^, SSC^lo^, Ly6C^lo^, F4/80^hi^), monocytes (CD11c^−^, CD11b^+^, SSC^lo^, Ly6C^lo^, F4/80^lo^), neutrophils (CD11c^−^, CD11b^+^, Ly6G^+^), basophils (FceR1^+^, CD117^−^), mast cells (FceR1^+^, CD117^+^), and NK cells (CD3e^−^, FceR1^−^, CD49b^+^, CD335^+^).

### 2.14. Luminex Analysis of Cytokines in Ex Vivo Restimulated Mouse Spleen Cell Supernatants

Spleens were collected from each mouse at the termination of the experiment aseptically and kept on ice in 0.1% BSA in PBS. Spleens were homogenized into a single-cell suspension and erythrocytes were lysed using sterile, distilled cold water. The lysed suspension was then filtered through a 70 µm cell strainer (Corning) and washed using Dulbecco’s Modified Eagle medium (Corning). Cells were measured and resuspended in 100 µL aliquots of 2 × 10^6^ cells in 96-well flat-bottom plates, and 0.5 µL of anti-IL4R (BD Biosciences) was added to each well. Splenocytes were stimulated with 10 µg/well of either *Ov*-103 or *Ov*-RAL-2 for 72 h at 37 °C. Supernatants were analyzed for cytokine concentrations using Milliplex Map Kit magnetic bead panels per the manufacturer’s protocols (EMDMillipore) and analyzed using a MAG-PIX Luminex multiplexing instrument (Luminex, Austin, TX). Concentrations of IL-33, IL-17F, IL-17A, IL-13, IL-10, IL-6, IL5, IL-4, IL-2, and IFN-γ were calculated using Milliplex Analyst software version 5.1 Flex (EMDMillipore).

### 2.15. Enzyme-Linked Immunosorbent Assay Analysis of Antigen-Specific Antibody in Mouse Serum

Serum was collected from each mouse at the termination of each experiment. Nunc MaxiSorp 96-well flat-bottom ELISA plates (Thermo Fisher Scientific, Waltham, MA, USA) were coated with 1 µg/mL of either *Ov*-103, *Ov*-RAL-2, or *Ov*-FUS-1 in 0.1 M carbonate buffer pH 9.6 for 12 h at 4 °C. All wells were washed 5× with 0.05% Tween 20 in TBS (TBS-T). Wells were then blocked using blocking buffer (2% BSA in TBS-T) for one hour at room temperature and then washed. Serum was diluted serially in blocking buffer and incubated for 90 min at 37 °C. Wells were washed five times with blocking buffer, and goat anti-mouse IgG1 (1:15,000); goat anti-IgG2a, IgG2b, and IgG2c (1:15,000 each); or goat anti-mouse IgE (1:8000) secondary horseradish peroxidase-conjugated antibodies (Southern Biotech, Birmingham, AL, USA) were added and incubated for 45 min at room temperature. All wells were washed five times with blocking buffer and 100 µL TMB solution (SeraCare, Gaithersburg, MD, USA) for 10 min for IgG1 and 15 min for IgG2abc or IgE at room temperature. Immediately after, 100 µL of TMB stop solution (SeraCare) was added and the optical density was measured at 450 nm using an iMark plate reader (BioRad, Hercules, CA, USA). Endpoint titers were calculated using SoftMax Pro version 6.5.1 software (Molecular Devices, San Jose, CA, USA). Minimum positive titers were determined by the lowest serum dilution from immunized mice with an optical density three times higher than the background. Endpoint titers of zero were assigned to samples with an optical density at background levels. While the ELISA assay was designed to detect IgG2abc subclasses, BALB/cByJ mice only express IgG2a and IgG2b [51] and, therefore, only the antibody titers for these two subclasses were reported.

### 2.16. Enzyme-Linked Immunosorbent Assay Analysis of Antigen-Specific Antibody in NHP Serum

Blood was collected from immunized NHPs on Days −13, 13, 27, 43, 57, 71, 85, 98/99, and 210. After being left to clot at 4 °C for 30 min, serum was collected. IgG antibody titers to *Ov*-103 and *Ov*-RAL-2 were measured by ELISA from pooled diffusion chamber fluid from each NHP, and serum was collected at various time points. CoStar 96-well ELISA plates (Corning) were coated with 50 μL of 1 μg/mL *Ov*-103 and *Ov*-RAL-2 antigens diluted in 0.05 M carbonate buffer pH 9.6 (Thermo Fisher Scientific, Waltham, MA, USA) and incubated overnight at 4 °C. Plates were washed with 1 × PBS (Life Technologies) + 0.05% Tween 20 between each step. Plates were blocked with 1 × PBS + 0.05% Tween 20% + 3% BSA for 1.5 h at 37 °C. Sera samples diluted to the appropriate concentration in assay buffer (1 × PBS + 0.05% Tween 20 + 1% BSA) were added to each well in a volume of 50 μL and incubated for two hours at 37 °C. Horseradish peroxidase-conjugated goat anti-monkey IgG (Invitrogen) was diluted to a working concentration in assay buffer, and 50 μL/well was added for an incubation period of one hour at 37 °C. Plates were developed in the dark for 15 min at room temperature with 50 μL/well of TMB solution (Seracare). The reaction was stopped by the addition of 25 μL/well of 2N H_2_SO_4_, and the optical densities were measured at 450 nm using a Glomax reader (Promega, Madison, WI, USA). Endpoint titers were calculated using GraphPad Prism software version 6.07 (San Diego, CA, USA). A cut-off value of 0.1 OD was used.

### 2.17. Statistics

Mouse experiments consisted of 5–6 mice per group, with the experiments performed at least twice with consistent results between experiments. Data from all experiments were combined for analysis. Data for larval survival were analyzed by multi-factorial analysis of variance with post hoc Fisher’s least significant difference testing in Systat v.11 (Systat Inc., Evanston, IL, USA). Probability values (*p*) of ≤ 0.05 were considered statistically significant. For the cytokine analyses, cytokine counts were rank-transformed prior to analysis. Each cytokine was analyzed separately using a linear regression model to analyze differences between cytokine values, depending on the comparison of interest. *p* values were adjusted for multiple testing to control the false discovery rate (FDR) [52]. Heat maps were created as a visual summary of the FDR-adjusted *p* values. All analyses were completed using SAS 9.4 and SAS/STAT 15.1 (SAS Institute, Cary, NC, USA). Plots and figures were generated using GraphPad Prism 9 (Dotmatics, Boston, MA, USA).

## 3. Results

### 3.1. Immunization of Mice with Advax-CpG-Adjuvanted Ov-103 and Ov-RAL-2 Combined Antigens or with Bivalent Ov-FUS-1 Protein Induces Rapid Larval Killing

**Figure 1:**
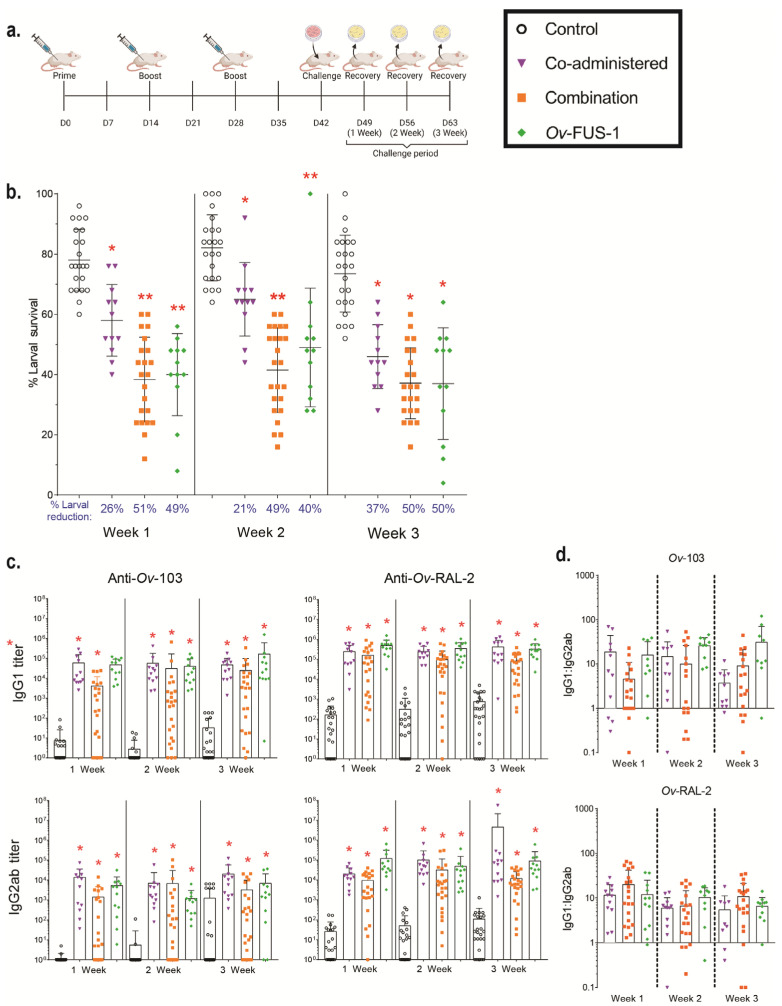
Immunization with combination and fusion *O. volvulus* vaccine antigens formulated with Advax-CpG induced protective immunity in mice. (**a**) BALB/cByJ mice were immunized with either *Ov*-103 and *Ov*-RAL-2 co-administered (*n* = 22–23), either in combination (*n* = 12), or as a fusion protein (*Ov*-FUS-1) (*n* = 12) formulated with the adjuvant Advax-CpG. Control mice (*n* = 22–23) received Advax-CpG-only injections. Injections were administered in a prime–boost–boost sequence two weeks apart. Two weeks post-final booster, diffusion chambers containing 25 *O. volvulus* infective third-stage larvae were implanted, followed by recovery one, two, or three weeks later. (**b**) Survival of *O. volvulus* L3 in diffusion chambers. Data are shown as mean percent larval survival, with individual mice represented as points. Error bars indicate standard deviations. Percent reduction in larval survival, when compared to control mice for each time point, is listed below the horizontal axis. (**c**) Antigen-specific IgG titers measured in the serum of control and immunized mice at the time of recovery. Data shown are mean titers, with individual mice represented as points. Error bars indicate standard deviations. IgG subclass is indicated on the vertical axis, and antigen specificity is indicated above the two associated plots. (**d**) Ratios of IgG1 to IgG2ab from the serum of individual mice at the time of recovery. Data shown are mean titer ratios, with individual mice represented as points. Error bars indicate standard deviations. IgG subclass is indicated on the vertical axis, and antigen specificity is indicated above the associated plots. Robust regression and outlier removal method with a false discovery rate of 1% was conducted using GraphPad Prism to eliminate 26 outliers. (**b**–**d**) * = *p* ≤ 0.05, indicating significant differences when comparing means to control animals within the time point. ** = *p* ≤ 0.05, indicating significant differences when comparing means to control animals and co-administered recipients within the time point.

A fusion of the *O. volvulus* antigens *Ov*-103 and *Ov*-RAL-2 (*Ov*-FUS-1) was generated in *Pichia pastoris* by expressing the two antigens in tandem with a 15-amino acid linker. BALB/cByJ mice were immunized with (A) “co-administered” antigens consisting of *Ov*-103 and *Ov*-RAL-2 injected into separate caudal thigh muscles, (B) “combination” antigens consisting of *Ov*-103 and *Ov*-RAL-2 premixed and injected together into each caudal thigh muscle, or (C) *Ov*-FUS-1 injected into each caudal thigh muscle. Vaccine antigens were formulated with Advax-CpG, and immunizations occurred in a prime–boost–boost sequence two weeks apart, whereas control animals were injected with Advax-CpG only. Two weeks after the final booster, mice received a challenge infection with *O. volvulus* L3 in diffusion chambers, which were removed after one, two, or three weeks (Figure 1a). The number of surviving larvae recovered from the diffusion chambers was significantly reduced in all immunized groups compared to control animals at all recovery time points. However, the level of protection, measured by the percent reduction in larval survival, varied based on the time point measured and the vaccine antigen formulation. One and two weeks post-challenge, larval survival was significantly lower in mice immunized with combination and *Ov*-FUS-1 vaccines compared to the co-administered vaccine. By three weeks post-challenge, all three vaccines induced equivalent reductions in larval survival. Combination antigens and *Ov*-FUS-1 induced similar levels of protection, ranging from a 40% to 51% reduction in larval survival across all time points. The co-administered vaccine induced the lowest levels of protective immunity, ranging from 21% at two weeks to a maximum reduction of 37% at three weeks (Figure 1b).

Immunized mice generated significantly greater *Ov*-103- and *Ov*-RAL-2-specific IgG1 and IgG2ab antibody titers compared to control animals, regardless of the vaccine formulation. The titers ranged from 3-fold higher in the combination-immunized mouse IgG2ab at three weeks post-challenge to 40,000-fold higher in the co-administered-immunized mouse IgG2ab at three weeks post-challenge (Figure 1c, Table S1). The majority of animals had higher IgG1 responses compared to IgG2ab. The ratio of IgG1 to IgG2ab ranged from 11 to 59 and did not vary significantly when comparing either the formulation or time point (Figure 1d).

These results demonstrate that the co-administered, combination, and *Ov*-FUS-1 vaccine antigens formulated with Advax-CpG are all capable of protecting mice from larval challenge and inducing significant IgG1 and IgG2ab antigen-specific antibody responses. However, the combination and *Ov*-FUS-1 formulations induced a protective response that resulted in more rapid killing of *O. volvulus* L3 compared to the co-administered formulation.

### 3.2. Ov-FUS-1 Formulated with Advax-CpG Induces Durable Protective Immunity in Mice

**Figure 2:**
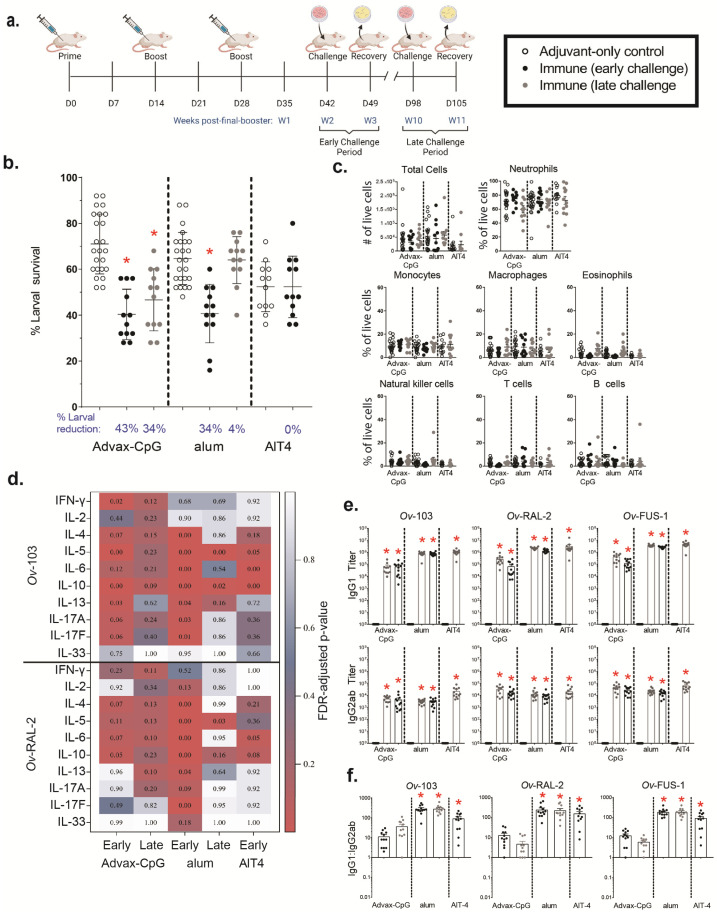
Immunization with *Ov*-FUS-1 formulated with the adjuvant Advax-CpG induced durable protective immunity in mice. (**a**) BALB/cByJ mice were immunized with *Ov*-FUS-1 formulated with either Advax-CpG (*n* = 12), alum (*n* = 12), or AlT4 (*n* = 12) as the adjuvant. Injections were administered in a prime–boost–boost fashion two weeks apart. Two weeks post-final booster, mice were challenged with diffusion chambers containing 25 *O. volvulus* L3, followed by recovery one week later. A separate group of mice was similarly primed and boosted but challenged 10 weeks post-final booster, followed by recovery one week later. Control mice (*n* = 24) received adjuvant-only injections that were combined from the early and late time points. (**b**) Survival of *O. volvulus* L3 in diffusion chambers. Data are shown as mean percent of surviving larvae, with individual mice represented as points. Error bars indicate standard deviations. Percent reduction in larval survival, when compared to control mice for each adjuvant group, is listed below the horizontal axis. (**c**) Total and differential counts of immune effector cells from diffusion chambers recovered from control and immunized mice measured by flow cytometry. Data are shown as mean cell counts or percent of total live cells, with individual mice represented as points. Error bars represent standard errors of the mean. (**d**) Heat map displaying cytokine responses produced by spleen cells, restimulated with antigen (*Ov*-103 and *Ov*-RAL-2), from immunized and control mice. Antigen and cytokines are displayed on the vertical axis corresponding to each row, and experimental groups are listed on the horizontal axis corresponding to each column. Data shown are *p* values generated via linear regression modeling comparing rank-transformed cytokine concentrations between control and immune mice within adjuvant groups. The *p* values are FDR-adjusted and progress from white (*p* = 1.00) to blue (*p* = 0.50) to red (*p* = 0.05). (**e**) Antigen-specific IgG titers measured in the serum of control and immunized mice at the time of recovery. Data shown are mean titers, with individual mice represented as points. Error bars indicate standard errors of the mean. IgG subclass is indicated on the vertical axis, and antigen specificity is indicated above the two associated plots. (**f**) Ratios of IgG1 to IgG2ab from the serum of individual mice at the time of recovery. Data shown are mean titer ratios, with individual mice represented as points. Error bars indicate standard deviations. IgG subclass is indicated on the vertical axis, and antigen specificity is indicated above the associated plots. (**b**–**e**) * = *p* ≤ 0.05, indicating significant differences when comparing means to control animals within the same adjuvant group. (**f**) * = *p* ≤ 0.05, indicating significant differences when comparing means to Advax-CpG early and late groups.

*Ov*-FUS-1 was selected for further characterization due to its efficacy in inducing protective immunity and the advantages of fusion proteins for future product and clinical development. To determine whether protective immunity could be enhanced through adjuvant selection, mice were immunized with *Ov*-FUS-1 formulated with three different adjuvants, Advax-CpG, alum, and AlT4. One group of mice was challenged two weeks post-final booster (early) and another at ten weeks post-final booster (late) to determine the durability of the vaccine-induced immune responses. Each challenge was followed one week later by larval recovery (Figure 2a). At the early recovery time point, a significant reduction in larval survival was detected only in mice immunized with *Ov*-FUS-1/Advax-CpG or alum but not with *Ov*-FUS-1/AlT4. Since significant larval killing was not observed in the *Ov*-FUS-1/AlT4-immunized mice at the early recovery time point, further evaluation of the *Ov*-FUS-1/AlT4 vaccine in mice was discontinued. At the late challenge time point, mice vaccinated with *Ov*-FUS-1/Advax-CpG retained protective immunity, with a 34% reduction in larval survival. However, mice vaccinated with *Ov*-FUS-1/alum did not retain protection, with only a 4% reduction in larval survival (Figure 2b).

Diffusion chamber fluid was analyzed using flow cytometry to determine the total number of live cells and the proportions of neutrophils, monocytes, macrophages, eosinophils, natural killer cells, T cells, B cells, mast cells, and basophils. All cell types, except for mast cells and basophils, were detected within the diffusion chambers recovered from control and vaccinated mice. The differences in the total number of live cells and proportion of the measured cell populations in the diffusion chambers were not significant when comparing immunized mice to adjuvant-only controls, regardless of the recovery time point. In control mice and vaccinated mice, neutrophils were the dominant cell population in the diffusion chamber, ranging from 60% to 79% of total live cells at both time points (Figure 2c).

A multiplex Luminex assay was used to measure cytokine responses in supernatants from mouse spleen cells restimulated ex vivo with *Ov*-103 and *Ov*-RAL-2. A vaccine was determined to induce a specific cytokine response if one or both of the antigens stimulated a significant increase in cytokine levels compared to appropriate adjuvant-only controls. At the early time point, *Ov*-FUS-1/Advax-CpG induced significant cytokine responses consisting of IFN-γ, IL-5, IL-10, and IL-13. *Ov*-FUS-1/alum induced IL-4, IL-5, IL-6, IL-10, IL-13, IL-17A, and IL-17F. *Ov*-FUS-1/AlT4 induced significant cytokine responses consisting of IL-5, IL-6, and IL-10. Only *Ov*-FUS-1/alum induced significant cytokine responses at the late time point consisting of IL-5 and IL-10 (Figure 2d, Table S2). While these results do not suggest that any single cytokine or combination of cytokines is required for protective immunity, this analysis indicates that *Ov*-FUS-1 induces a complex variety of cytokine responses to both antigens, depending on the adjuvant used in the vaccine.

Serum was collected at the early and late time points and *Ov*-103-, *Ov*-RAL-2-, and *Ov*-FUS-1-specific IgG1 and IgG2ab responses were measured. All immunized mice, regardless of the formulation, had significant increases in IgG1 and IgG2ab antibody titers against *Ov*-103, *Ov*-RAL-2, and *Ov*-FUS-1 antigens at the early time point, as did mice vaccinated using Advax-CpG and alum at the late time point (Figure 2e, Table S3). Antigen-specific IgG1 titers were higher than IgG2ab titers in all mice when compared within adjuvant groups. Alum- and AlT4-formulated vaccines induced a significantly higher ratio of IgG1:IgG2ab titers compared to Advax-CpG, regardless of the recovery time point. The IgG1:IgG2ab ratios across all time points and antigen specificities ranged from 5 to 37 for Advax-CpG, 186 to 288 for alum, and 93 to 159 for AlT4 (Figure 2f). There was no detectable antigen-specific serum IgE at either time point in any of the immunized mice.

### 3.3. Passive Transfer of Sera from Mice Immunized with Ov-FUS-1 and Advax-CpG Protects Naïve Mice

Naïve mice were challenged with L3 within the diffusion chambers and received a transfer of serum from naïve or immunized mice into the subcutaneous pocket where the diffusion chamber was implanted to determine whether protective immunity was a function of vaccine-induced serum factors. Serum from mice immunized with *Ov*-FUS-1/Advax-CpG was collected pre- and post-challenge with L3 in the diffusion chambers. Passive transfer of serum from both groups of immunized mice resulted in a significant reduction in larval survival compared to mice that received naïve serum. Passive transfer with pre-challenge immune sera resulted in a 32% reduction in larval (mean larval survival 49 ± 18%, *n* = 15), and passive transfer of post-challenge sera resulted in a significant reduction in larval survival of 29% (mean larval survival 52 ± 14%, *n* = 6). Thus, exposure of immunized mice to *O. volvulus* larvae in the diffusion chambers did not enhance the protective efficacy of their serum. Based on the equivalent levels of protection from both pre- and post-challenge immune sera, the two serum collection time points were subsequently used interchangeably and referred to as “*Ov*-FUS-1/Advax-CpG early immune serum”.

**Figure 3:**
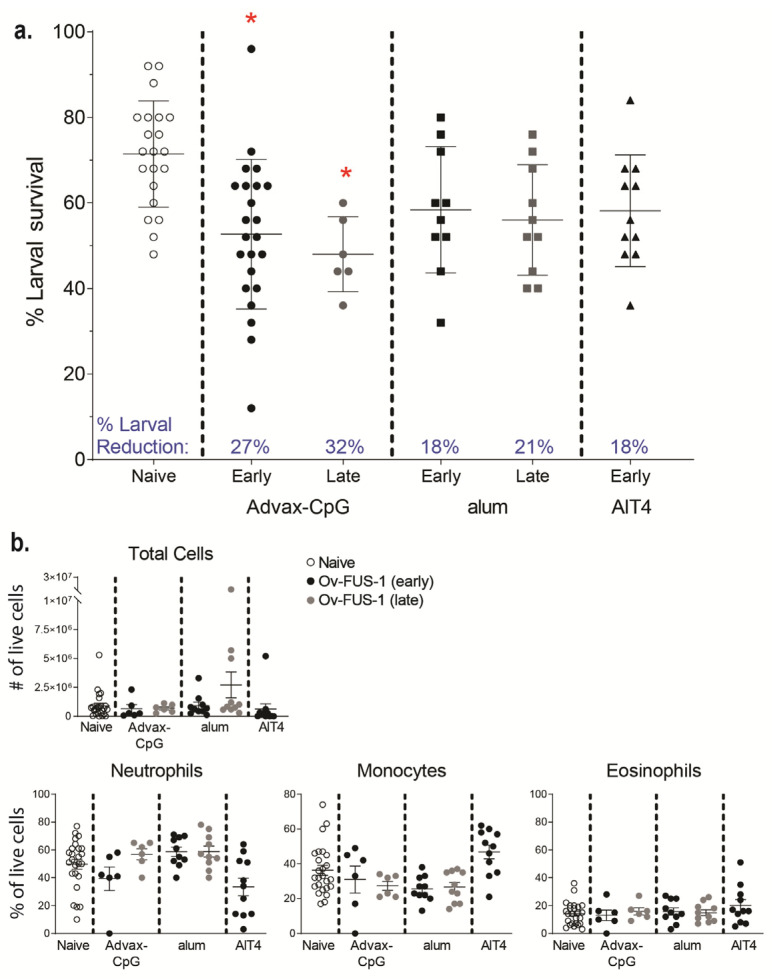
Durable protection can be transferred with serum from mice immunized with *Ov*-FUS-1 and Advax-CpG, regardless of exposure to challenge infection. (**a**) Larval survival following passive transfer of serum from mice immunized with *Ov*-FUS-1 and either Advax-CpG (early *n* = 23, late *n* = 6), alum (early *n* = 10, late *n* = 10), or AlT4 (*n* = 11) as the adjuvant. Serum was collected at the time of recovery following the early and late challenge period. Control mice received transfer of naïve mouse serum (*n* = 21), which were combined for early and late time points. Diffusion chambers were recovered one week after implantation and remaining live larvae were counted to determine larval survival. Data are shown as mean percent larval survival, with individual mice represented as points. Error bars indicate standard deviations. Percent reduction in larval survival, when compared to control mice, is listed above the horizontal axis. (**b**) Total and differential counts of immune effector cells from diffusion chambers recovered from control and immunized mice were measured by microscopy. Data are shown as mean cell counts or percent of total live cells, with individual mice represented as points. Error bars represent standard errors of the mean. (**a**,**b**) * = *p* ≤ 0.05, indicating significant differences when comparing means to control animals within the same adjuvant group.

Serum was pooled from mice immunized with *Ov*-FUS-1 formulated with either Advax-CpG, alum, or AlT4 as adjuvants at the early and late time points and passively transferred into naïve mice. Passive transfer of serum collected from *Ov*-FUS-1/Advax-CpG-immunized mice from the early time point resulted in a 27% reduction in larval survival, and from the late time point, a 32% reduction in survival. Neither *Ov*-FUS-1/alum early or late serum nor *Ov*-FUS-1/AlT4 early serum transferred significant protection into mice (Figure 3a). Cells collected from diffusion chambers recovered following passive serum transfer were differentiated morphologically. The total number of cells and the proportions of neutrophils, monocytes, and eosinophils were consistent across all groups. Similar to active vaccination (Figure 2c), neutrophils were the dominant cell type in the parasite microenvironment, ranging from 39% to 59% of total live cells. The only exception was with serum from mice immunized with *Ov*-FUS-1/AlT4, where monocytes were the dominant cell type, constituting 47% of total live cells (Figure 3b).

### 3.4. Ov-FUS-1 Formulated with Three Different Adjuvants Induces High-Titer Antibody Responses in NHPs

**Figure 4:**
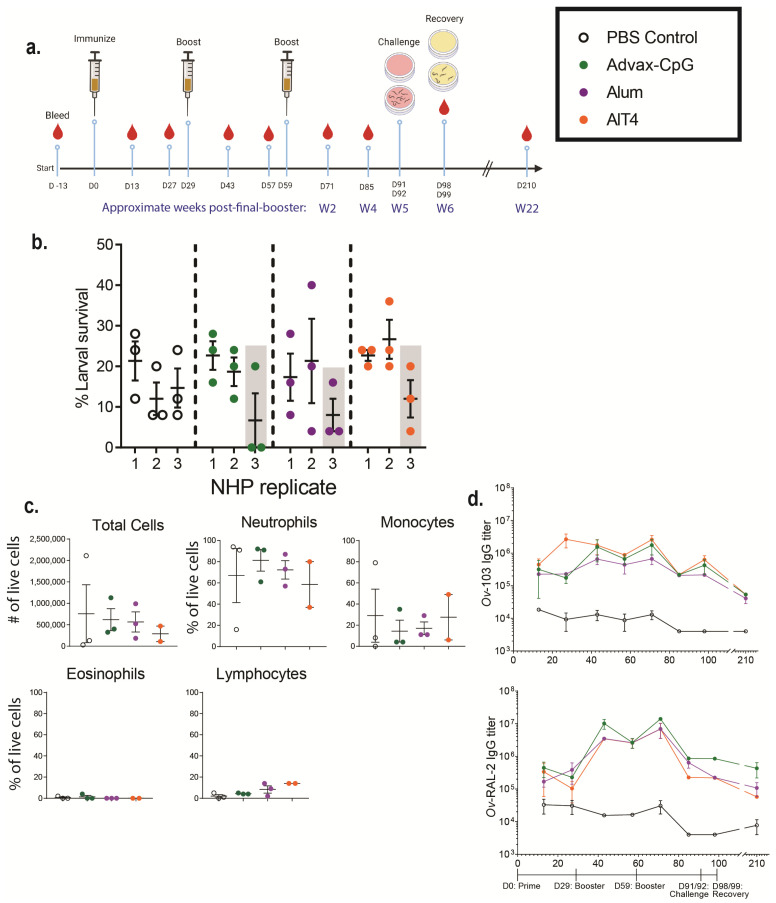
*Ov*-FUS-1 is immunogenic in NHPs, regardless of adjuvant formulation. (**a**) NHPs were immunized with *Ov*-FUS-1 and either Advax-CpG (*n* = 3), alum (*n* = 3), or AlT4 (*n* = 3) as the adjuvant. Control NHPs received PBS-only injections (*n* = 3). Injections were administered in a prime–boost–boost fashion 30 days apart. NHPs were challenged with diffusion chambers containing 25 *O. volvulus* L3, 32 and 33 days post-final booster, followed by recovery 7 days later. Serum was collected from NHPs every two weeks up to Day 99 and again at Day 210. (**b**) Larval survival measured in diffusion chambers recovered from NHPs. Data are shown as mean larval survival within individual NHPs. Larval survival within individual diffusion chambers (Three per NHP) is represented by points. Shaded columns indicate one NHP from each group with the greatest reduction in larval survival. (**c**) Total and differential counts of immune effector cells from diffusion chambers recovered from control and immunized NHPs measured by microscopy. Data are shown as mean cell counts or percent of total live cells, with samples from individual NHPs represented as points. Error bars represent standard errors of the mean. (**d**) Antigen-specific IgG titers measured in the serum of control and immunized NHPs throughout the trial. Data shown are mean titers with error bars indicating standard errors of the mean. IgG subclass and antigen specificity are indicated on the vertical axis. Time course indicating prime, boosters, challenge, and recovery is shown below the plots.

NHPs received three immunizations with *Ov*-FUS-1 formulated with either Advax-CpG, alum, or AlT4 as adjuvants approximately four weeks apart. Control NHPs received PBS injections, and each experimental group consisted of three NHPs. The animals were monitored for any adverse events following vaccination and there were no changes in temperature, appetite, or activity. In addition, there was no evidence of inflammation or lesions at the injection site. Eight diffusion chambers were implanted in each NHP four weeks post-final booster. Five diffusion chambers contained *O. volvulus* L3, of which three were analyzed and reported in this study, and three diffusion chambers contained media alone, of which two were analyzed and reported in this study. Diffusion chambers were removed one week after implantation to determine larval survival and cell infiltration (Figure 4a). *O. volvulus* larval survival in the three PBS-treated NHPs had a mean of 16%, whereas the mean larval survival ranged from 16 to 20% across all immunized NHPs, regardless of adjuvant. Each adjuvant vaccine group had one NHP with reduced larval survival. In the *Ov*-FUS-1/Advax-CpG group, one NHP had a mean reduction in larval survival of 56%; in the *Ov*-FUS-1/alum group, one NHP had a mean reduction of 50%; and in the *Ov*-FUS-1/AlT4 group, one NHP had a mean reduction of 25% compared to the mean survival in the PBS control NHPs (Figure 4b).

The recruitment of effector immune cells was measured by microscopy to quantify the number of cells within the diffusion chambers at the time of recovery. Total cells and the proportions of immune cell subsets were consistent across all vaccination groups and control NHPs. Neutrophils were the dominant subset, ranging from 59% in the AlT4 group to 81% in the Advax-CpG group (Figure 4c).

Serum was collected from NHPs every 14 days for 98 days, with an additional sample collected at 210 days following the initial prime immunization. Antigen-specific IgG antibody titers were measured for both *Ov*-FUS-1 component antigens, *Ov*-103 and *Ov*-RAL-2. Immunized NHPs, regardless of adjuvant, developed elevated IgG responses to *Ov*-103 and *Ov*-RAL-2. Two weeks post-final booster, on Day 71, the fold increase in *Ov*-103 and *Ov*-RAL-2-specific IgG in immunized mice compared to controls ranged from 51 to 460. Approximately 22 weeks post-final booster on Day 210, there was a 7–55-fold increase in IgG titers. Although there was a consistent increase in antigen-specific IgG titers in the immunized NHP, due to the small sample size and large range in the responses, statistical significance was achieved at only limited time points. There was no significant difference in IgG titers between adjuvant groups at any of the measured time points (Figure 4d, Table S4). Antigen-specific antibodies were measured in the diffusion chamber fluid at the time of parasite recovery. Both anti-*Ov*-103 and anti-*Ov*-RAL-2 IgG were detected in the diffusion chambers of all immunized NHPs, regardless of adjuvant or whether the diffusion chambers contained L3. Antigen-specific IgG titers in the diffusion chambers did not differ from the titers in the serum of the immunized NHPs (Table S5).

### 3.5. Passive Transfer of Serum from NHPs Immunized with Ov-FUS-1 and Advax-CpG into Naïve Mice Is Protective against O. volvulus L3 Challenge

**Figure 5:**
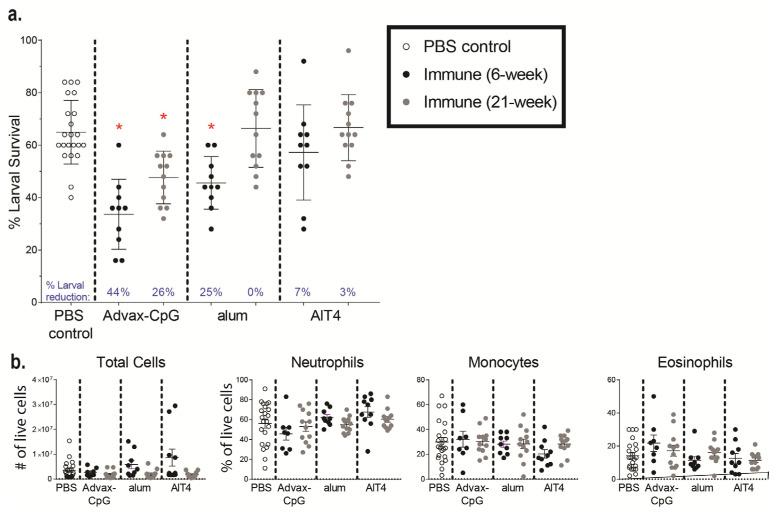
Durable protection can be transferred to mice with serum from NHPs immunized with *Ov*-FUS-1 and Advax-CpG. (**a**) Larval survival following passive transfer of pooled serum from NHPs immunized with *Ov*-FUS-1 and either Advax-CpG (*n* = 10), alum (*n* = 10), or AlT4 (*n* = 10) as the adjuvant into naïve mice. Control mice received transfer of PBS-treated NHP serum (*n* = 22). Serum was collected from NHPs 6 or 22 weeks post-final booster. Mice received serum at the time of challenge with *O. volvulus* L3 within diffusion chambers, which were recovered one week after implantation, and remaining larvae were counted to determine larval survival. Data are shown as mean percent larval survival, with individual mice represented as points. Error bars indicate standard deviations. Percent reduction in larval survival, when compared to control mice, is listed above the horizontal axis. (**b**) Total and differential counts of immune effector cells from diffusion chambers recovered from control and immunized mice measured by microscopy. Data are shown as mean cell counts or percent of total live cells, with individual mice represented as points. Error bars represent standard errors of the mean. * = *p* ≤ 0.05, indicating significant differences when comparing means to control animals.

Transfer of NHP serum into mice was conducted to determine whether the immunized NHPs developed a functional antigen-specific antibody response capable of killing *O. volvulus* larvae in vivo. Naïve mice were challenged with *O. volvulus* L3 within the diffusion chambers and simultaneously received pooled serum collected 6 weeks and 22 weeks post-final booster from PBS-treated NHPs or NHPs immunized with *Ov*-FUS-1 formulated with either Advax-CpG, alum, or AlT4. All mice received a second dose of serum three days post-challenge, and the diffusion chambers were recovered seven days post-challenge to determine larval survival. Mice treated with serum from NHPs immunized with *Ov*-FUS-1/Advax-CpG collected at the 6-week time point had a significant reduction of 44% in larval survival, and serum from the 22-week time point resulted in a significant 26% reduction. Mice treated with serum from NHPs immunized with *Ov*-FUS-1/alum had a significant reduction in larval survival of 25% but only with serum collected at six weeks post-final booster (Figure 5a). Serum from *Ov*-FUS-1/AIT4 vaccinated NHPs did not transfer protection. Regardless of the vaccine formulation or collection time point, neutrophils, monocytes, and eosinophils were present in the diffusion chambers, and the numbers of each cell type were consistent across all groups. Neutrophils were the dominant immune cell subset, constituting 46–68% of total live cells (Figure 5b). These results suggest that the antibody response in NHPs induced by immunization with *Ov*-FUS-1 formulated with either Advax-CpG or alum can transfer protection against *O. volvulus* challenge, but durable protection is only transferable with sera from *Ov*-FUS-1/Advax-CpG vaccinated NHPs.

## 4. Discussion

In the present study, we identified a vaccine formulation that induces a consistent, robust, and durable protective immune response against *O. volvulus* larvae in mice and NHPs. An established mouse model was used to evaluate the efficacy of vaccines composed of different antigen and adjuvant combinations and to characterize the induced protective immune responses. Parallel studies were conducted in NHPs to validate the results from mice and support the translation of the *O. volvulus* vaccine to humans. These studies demonstrate that *Ov*-FUS-1 formulated with the adjuvant Advax-CpG is immunogenic, safe, and capable of inducing durable protective immunity in both mice and NHPs. Additionally, it was shown that vaccine-induced protective immunity is likely dependent on antigen-specific immunoglobulins in both mice and NHPs.

The optimal antigen formulation was identified in mice by comparing the protective immune responses to the two vaccine antigens, *Ov*-103 and *Ov*-RAL-2. They were co-administered as two separate vaccine injections, combined into a single injection and as a fusion product of the two antigens, and tested at one-, two-, and three-week challenge periods. The fusion antigen, *Ov*-FUS-1, and the combination formulation induced maximum protection one week post-challenge, indicating that these formulations can induce larval killing at a faster rate compared to the co-administered vaccine antigens. This finding suggests that the target of the immune response is the L3, as roughly 85% of larvae have been shown to either molt or synthesize the fourth-stage larval cuticle after seven days in diffusion chambers in vivo [10]. The 40–51% reduction in larval survival induced by these vaccine formulations is consistent with previous studies in genetically diverse mice, evaluating the combination vaccine one week post-challenge [16]. Furthermore, the induction of protective immunity by *Ov*-FUS-1 with Advax-CpG supports the previous study in which mice immunized using a fusion of *Ov*-103 and *Ov*-RAL-2, with a different peptide linker sequence and alum as the adjuvant, induced protective immunity in BALB/cByJ mice. The reduction in larval survival induced by the *Ov*-FUS-1/Advax-CpG vaccine was superior to the 21% reduction induced by the previous fusion protein adjuvanted with alum [12]. Despite targeting the same antigens and being formulated with the same adjuvant, the bilateral co-administration of *Ov*-103 and *Ov*-RAL-2 resulted in a slower rate of protection compared to the combination and *Ov*-FUS-1 formulations. The slower rate of larval killing by the co-administered vaccine may be due to either the slower recruitment of factors required for killing or a reduced magnitude of effector responses, resulting in slower death of the larvae. While all three antigen presentations were effective in inducing equivalent levels of protective immunity three weeks post-immunization, *Ov*-FUS-1 was selected for further pre-clinical development based on the speed of killing the larvae and the higher antibody responses in *Ov*-FUS-1-immunized mice compared to combination-immunized mice. Additionally, a fusion could be more cost-efficient for large-scale manufacturing and administration in future clinical trials, as well as the vaccination of vulnerable populations [19,20,21]. Fusion antigens have been shown to be effective against cancer and a variety of pathogens, including bacteria, viruses, and parasites [19,53,54,55]. A fusion of the *O. volvulus*-orthologous antigens *Bm*-103 and *Bm*-RAL-2 formulated with alum induced protective immunity against *B. malayi* in gerbils, reducing the worm burden and fecundity of adult females [23]. Similar results were also seen when the fusion protein, r*Bm*HAXT, was used to immunize against infection with *B. malayi* in mice, gerbils, and NHPs [24,25,26,27].

The adjuvant selection was accomplished by comparing the efficacy in mice of *Ov*-FUS-1 formulated with either Advax-CpG, alum, or AlT4. Only *Ov*-FUS-1 with Advax-CpG induced durable protective immunity. This was evident from a significant reduction in larval survival at both 3 and 11 weeks post-final booster with the Advax-CpG-adjuvanted vaccine compared to the alum- and AlT4-adjuvanted vaccines. *Ov*-FUS-1/alum induced protective immunity 3 weeks post-final booster but not at 11 weeks post-final booster. For future human use, including in children, it is important to have an adjuvant that has a track record of human safety. For this reason, our lead adjuvant is Advax-CpG which, in addition to showing promising efficacy in our animal models, is already included as an adjuvant in a recombinant spike protein COVID-19 vaccine, SpikoGen^®^, that was shown to be safe and effective in a Phase 3 trial [56].

Parasite survival in diffusion chambers implanted in control NHPs was significantly lower than that in mice. Previous studies reported that larval *O. volvulus* had better survival in diffusion chambers implanted in mice compared to NHPs, including primate species susceptible to complete infection [10]. Due to the low baseline survival of *O. volvulus* larvae in NHPs, no significant reduction in larval survival was observed in the immunized animals. Despite this, the Advax-CpG- and alum-adjuvant vaccinated NHP groups had one NHP with a 50% or greater reduction in larval survival, suggesting that a protective immune response was induced in these NHPs. The variation within the responses may be explained by genetic diversity within the outbred NHPs. Similarly, studies in collaborative cross-recombinant inbred intercross mouse lines immunized against *O. volvulus* demonstrated that one of the lines with a strong innate protective response did not demonstrate enhanced killing by the adaptive immune response [16].

Cytokine responses from mouse spleen cells, restimulated ex vivo, were measured to characterize the systemic immune response to vaccination with *Ov*-FUS-1 and either Advax-CpG, alum, or AlT4. Various cytokine responses were observed, suggesting that adjuvant formulation modulates the antigen-induced splenic cytokine responses. These results are similar to previous studies using different *O. volvulus* vaccine formulations with various adjuvants [15,16]. *Ov*-FUS-1/Advax-CpG induced a mixed Th1/Th2 response as indicated by elevated Th1 cytokines (IFN-γ) and Th2 cytokines (IL-4, IL-5) three weeks post-final booster. The current findings are also in accordance with previous studies using both co-administered *Ov*-103 and *Ov*-RAL-2 with Advax-CpG in mice [16] and other Advax-based vaccines [30,31,35,57,58]. A balanced Th1/Th2 response was also associated with vaccine-induced protective immunity against *B. malayi* [24,25,26]. *Ov*-FUS-1 with both alum and AlT4 induced Th2-biased responses with elevated IL-4 and IL-5 and an absence of elevated IFN-γ. Helper T cell bias was corroborated by the ratios of IgG1:IgG2ab induced following vaccination in mice, where *Ov*-FUS-1/Advax-CpG had a more balanced ratio compared to *Ov*-FUS-1 with alum or AlT4 [59]. These results suggest that the balance of Th1/Th2 responses in immunized mice is important for protective immunity against *O. volvulus*.

While *Ov*-FUS-1/Advax-CpG was the only formulation to induce durable protective immunity in mice, all immunized mice and NHPs developed high antigen-specific IgG titers. These data suggest that *Ov*-FUS-1 is immunogenic with all three adjuvants tested in this study. Similar observations were described in cows following vaccination with co-administered *Ov*-103 and *Ov*-RAL-2 formulated with alum, Advax-CpG, and Montanide adjuvants [17]. Antigen-specific IgE was not detected in serum collected from mice three weeks post-final booster, regardless of adjuvant. Antigen-specific IgE has been implicated in severe adverse reactions following vaccination with the *Na*-ASP-1 hookworm vaccine [60]. The absence of vaccine-induced IgE reduces the risk of adverse reactions following vaccination with *Ov*-FUS-1. IgE was shown to be integral in the protective immune response to *O. volvulus* induced by irradiated *O. volvulus* L3 [61]. Therefore, the mechanism of protective immunity induced by irradiated larvae, based on IgE, is integrally different from the IgG-associated immunity induced by *Ov*-FUS-1/Advax-CpG. The absence of an antigen-specific IgE response after vaccination may also alleviate any potential interaction with microfilariae that could result in pathological consequences. The observation that young children lack functional antigen-specific IgE responses against *Ov*-103 and *Ov*-RAL-2 suggests that the vaccine will be safe and effective in children [62].

The development of protective immunity induced by vaccination was corroborated by the passive transfer of protection with immune serum from mice and NHPs into naïve mice. The detection of anti-*Ov*-103 and anti-*Ov*-RAL-2 antibodies in the diffusion chamber fluid at the same titer as in the associated serum sample indicates that the antibodies diffuse from the serum into the parasite microenvironment. Only *Ov*-FUS-1/Advax-CpG generated a protective antibody response sufficient to mediate statistically significant transferable protection at both the early and late time points. Furthermore, serum from both vaccinated mice and NHPs was capable of passively transferring immunity at both time points. Similar results were obtained in mice through the passive transfer of serum from immunized cynomolgus NHPs that protected against liver-stage *Plasmodium falciparum* [58] or purified IgG from vaccinated baboons that protected against *Schistosoma mansoni* [63]. These data suggest that serum antibodies mediate protective immunity, which is in accordance with a prior study that demonstrated that AID^−/−^ mice lacking the class-switched antibody do not develop protective immunity following immunization with individual *Ov*-103 and *Ov*-RAL-2 antigens formulated with alum [18]. It is important to note that there was no correlation between antigen-specific antibody titers and the ability of serum to transfer immunity. Differences in antibody effector function mediated, for example, by different Fc glycosylation patterns induced by different adjuvants, may be responsible for the discrepancy between the efficacy and antibody titer [64]. Alternatively, responses against certain protective epitopes on the antigens are preferentially induced based on the adjuvant used. This hypothesis is supported by previous studies, demonstrating that the selection of adjuvants can modulate epitope specificity, resulting in varying levels of protection based on the adjuvant used [65,66,67].

Contact between cells and parasites was shown to be essential in the killing process induced by vaccination with either *Ov*-103 or *Ov*-RAL-2 formulated with alum [18]. All of the measured cell populations remained consistent across the control and immunized animals in both models. The fact that the passive transfer of immunity with serum could protect mice from *O. volvulus* challenge at the same magnitude as active immunization suggests that humoral factors collaborate with innate cells, independent of their activation status. In both mice and NHPs, neutrophils were consistently the most abundant immune cell within the parasite microenvironment across the control and immunized animals, suggesting that they may play an important role in the larval killing mechanism. Neutrophils have been shown to actively participate in the immune control of filarial worm infections [68] and, therefore, may be the effector cells that collaborate with antibodies to kill worms following vaccination.

This study demonstrated that all tested vaccine formulations were immunogenic and safe in mice and NHPs. However, only *Ov*-FUS-1/Advax-CpG induced durable protective immunity, as evidenced by the killing of larvae in vivo in vaccinated mice and the passive transfer of immunity with serum from both vaccinated mice and NHPs. In both the mouse and NHP experiments, reductions in larval survival ranged from 27% to 50% across the immunization and passive transfer studies. Based on an *O. volvulus* transmission model, a vaccine with an efficacy of approximately 50% against L3s delivered to infants would greatly reduce the microfilarial burden in children, as well as transmission and the likelihood of developing severe disease symptoms [69]. Therefore, partial protection induced by the *Ov*-FUS-1/Advax-CpG vaccine would potentially have a complementary effect, when integrated with other control measures, toward reaching the World Health Organization’s goal of eliminating onchocerciasis by 2030 [70]. An effective vaccine complementing drugs that target the prevalence of microfilariae in the skin would also decrease the burden of onchocerciasis-associated disease in areas of persistent high transmission. The parallel efficacy of *Ov*-FUS-1 formulated with the adjuvant Advax-CpG in mice and NHPs supports further product development of this vaccine formulation and its translation for use in a first-in-human phase one clinical trial to initiate its clinical development plan.

## Data Availability

The data that support the findings of this study are available in the article and the Supplementary Materials, as well as from the corresponding author upon request. Additional details on Advax-CpG and other NIH-supported adjuvants can be accessed through the NIAID Vaccine Adjuvant Compendium database via the following link https://vac.niaid.nih.gov. The content is solely the responsibility of the authors and does not necessarily represent the official views of the National Institutes of Health.

## Supplementary Materials

The following supporting information can be downloaded at https://www.mdpi.com/article/10.3390/vaccines11071212/s1. Table S1: Antigen-specific antibody titers from mice immunized with co-administered, combination, or *Ov*-FUS-1 and the adjuvant Advax-CpG; Table S2: Splenic cytokine responses from mice immunized with *Ov*-FUS-1 and either Advax-CpG, alum, or AlT4 as the adjuvant; Table S3: Antigen-specific antibody titers from mice immunized with *Ov*-FUS-1 and either Advax-CpG, alum, or AlT4 as the adjuvant; Table S4: Antigen-specific antibody titers from NHPs immunized with *Ov*-FUS-1 and either Advax-CpG, alum, or AlT4 as the adjuvant; Table S5: Antigen-specific antibody titers compared between serum and diffusion chamber fluid from NHPs immunized with *Ov*-FUS-1 and either Advax-CpG, alum, or Alt4 as the adjuvant.
