# Supplementary material for: Adjuvanted Fusion Protein Vaccine Induces Durable Immunity to Onchocerca volvulus in Mice and Non-Human Primates
Source: Vaccines (Basel). 2023 Jul 6;11(7):1212. doi: 10.3390/vaccines11071212 (PMC10385774; doi:10.3390/vaccines11071212)
Supplement: Supplementary file 1 [file vaccines-11-01212-s001.zip › vaccines-2470717-supplementary.pdf]

Table S1. Antigen-specific antibody titers from mice immunized with co-administered, combination or *Ov*-FUS-1 and the adjuvant Advax-CpG. Serum was collected from immunized mice at the time of recovery following one-, two- and three-week challenge periods. IgG1 and IgG2ab specific for either *Ov*-103 or *Ov*-RAL-2 were measured in the serum using ELISA. Data shown are mean titers ± standard deviations. * p value ≤ 0.05 when compared to Advax-CpG-only controls at the same time point.

**Weeks post-challenge**	**Anti-*Ov*-103**			
	**IgG1**			
	**Control**	**Co-administered**	**Combination**	***Ov*-FUS-1**
**1 Week**	7 ± 18	**61,757 ± 91,652 ***	**4395 ± 7822 ***	**49,911 ± 41,184 ***
**2 Week**	3 ± 5	**58,848 ± 116,527 ***	**32,058 ± 134,823 ***	**41,584 ± 51,714 ***
**3 Week**	34 ± 64	**49,441 ± 50,380 ***	**25,949 ± 70,617 ***	**171,425 ± 438,341 ***
	**IgG2ab**			
	**Control**	**Co-administered**	**Combination**	***Ov*-FUS-1**
**1 Week**	1 ± 1	**14,627 ± 21,521 ***	**1475 ± 3196 ***	**5594 ± 8805 ***
**2 Week**	6 ± 23	**7,347 ± 16,382 ***	**7200 ± 23,080 ***	**1251 ± 1725 ***
**3 Week**	1,305 ± 2,382	**21,024 ± 36,046 ***	**3378 ± 6562 ***	**7275 ± 12,906***
	**Anti-*Ov*-RAL-2**			
	**IgG1**			
	**Control**	**Co-administered**	**Combination**	***Ov*-FUS-1**
**1 Week**	171 ± 287	**255,598 ± 243,842 ***	**165,257 ± 240,264 ***	**510,494 ± 398,289 ***
**2 Week**	329 ± 795	**254,904 ± 195,507 ***	**100,479 ± 155,982 ***	**364,215 ± 319,403 ***
**3 Week**	809 ± 1,226	**437,048 ± 443,877 ***	**86,816 ± 97,463 ***	**342,764 ± 263,447 ***
	**IgG2ab**			
	**Control**	**Co-administered**	**Combination**	***Ov*-FUS-1**
**1 Week**	28 ± 49	**20,956 ± 19,661 ***	**9884 ± 11,713 ***	**126,512 ± 187,605 ***
**2 Week**	53 ± 107	**107,379 ± 188,258 ***	**33,664 ± 81,982 ***	**52,093 ± 102,644 ***
**3 Week**	116 ± 259	**4,862,067 ± 16,569,576 ***	**12,375 ± 13,917 ***	**96,311 ± 169,425 ***

Table S2. Splenic cytokine responses from mice immunized with *Ov*-FUS-1 and either Advax-CpG, alum or AlT4 as the adjuvant. Spleens were collected from mice at the early and late recovery time points and restimulated with either *Ov*-103 (top) or *Ov*-RAL-2 (bottom) antigens. Supernatants from restimulated spleens were analyzed using a multiplex Luminex assay. Data are shown as mean concentrations (pg/mL) ± standard deviations. Fold differences depicted in red below values from control and immune mice for each cytokine at each time point. * p value ≤ 0.05 when compared to appropriate adjuvant-only controls at the same time point.

	***Ov*-103**	**IFN-γ**	**IL-2**	**IL-4**	**IL-5**	**IL-6**	**IL-10**	**IL-13**	**IL-17A**	**IL-17F**	**IL-33**
early	**Advax-CpG Control**	498 ± 1036	103 ± 70	108 ± 136	43 ± 37	209 ± 256	28 ± 2	198 ± 75	161 ± 75	77 ± 87	111 ± 30
	**Advax-CpG Immune**	4840 ± 1852	89 ± 39	425 ± 160	494 ± 473	1381 ± 1787	188 ± 142	313 ± 173	744 ± 791	352 ± 363	87 ± 9
		**10***	**1**	**4**	**12***	**7**	**7***	**2***	**5**	**5**	**1**
	**Alum Control**	1473 ± 2078	272 ± 246	136 ± 102	75 ± 53	469 ± 469	51 ± 25	130 ± 101	385 ± 281	200 ± 192	394 ± 85
	**Alum Immune**	1178 ± 900	313 ± 276	871 ± 521	792 ± 736	5513 ± 3750	301 ± 374	676 ± 827	464 ± 438	186 ± 141	464 ± 133
		**1**	**1**	**6 ***	**11 ***	**12 ***	**6 ***	**5 ***	**1 ***	**1 ***	**1**
	**Alt4 Control**	1416 ± 2051	173 ± 140	296 ± 281	112 ± 81	497 ± 507	33 ± 12	164 ± 89	195 ± 201	111 ± 207	178 ± 175
	**Alt4 Immune**	1737 ± 2359	149 ± 127	355 ± 208	876 ± 1319	2570 ± 2615	387 ± 431	836 ± 1047	565 ± 823	284 ± 597	415 ± 246
		**1**	**1**	**1**	**8 ***	**5 ***	**12 ***	**5**	**3**	**3**	**2**
late	**Advax-CpG Control**	364 ± 638	121 ± 78	95 ± 120	44 ± 82	203 ± 357	ND	48 ± 20	ND	ND	ND
	**Advax-CpG Immune**	798 ± 833	198 ± 103	226 ± 178	64 ± 50	489 ± 634	33 ± 12	89 ± 78	53 ± 9	67 ± 15	ND
		**2**	**2**	**2**	**1**	**2**	**x**	**2**	**x**	**x**	**x**
	**Alum Control**	711 ± 1141	155 ± 87	282 ± 450	94 ± 186	1845 ± 3620	253 ± 254	557 ± 620	71 ± 47	ND	ND
	**Alum Immune**	1360 ± 1641	189 ± 64	383 ± 256	298 ± 295	793 ± 533	68 ± 93	283 ± 592	60 ± 65	58 ± 55	ND
		**2**	**1**	**1**	**3***	**-2**	**-4***	**-2**	**1**	**x**	**x**

	***Ov*-RAL-2**	**IFN-γ**	**IL-2**	**IL-4**	**IL-5**	**IL-6**	**IL-10**	**IL-13**	**IL-17A**	**IL-17F**	**IL-33**
early	**Advax-CpG Control**	594 ± 1135	316 ± 349	253 ± 369	85 ± 114	480 ± 636	47 ± 19	252 ± 205	227 ± 157	104 ± 34	314 ± 120
	**Advax-CpG Immune**	3682 ± 2999	393 ± 413	628 ± 559	428 ± 456	1853 ± 1536	181 ± 168	379 ± 362	390 ± 222	191 ± 147	393 ± 152
		**6**	**1**	**2**	**5**	**4**	**4 ***	**2**	**2**	**2**	**1**
	**Alum Control**	457 ± 525	161 ± 124	78 ± 55	39 ± 26	125 ± 100	19 ± 7	57 ± 24	101 ± 48	24 ± 25	ND
	**Alum Immune**	537 ± 318	316 ± 208	457 ± 270	336 ± 330	2804 ± 2872	113 ± 131	215 ± 282	215 ± 175	57 ± 36	137 ± 43
		**1**	**2**	**6 ***	**9 ***	**23 ***	**6 ***	**4 ***	**2**	**2 ***	**2**
	**Alt4 Control**	1670 ± 2882	532 ± 514	695 ± 853	256 ± 283	1339 ± 1442	63 ± 59	485 ± 471	319 ± 157	119 ± 117	310 ± 197
	**Alt4 Immune**	1656 ± 1023	423 ± 187	931 ± 643	864 ± 546	5740 ± 3113	419 ± 235	756 ± 269	486 ± 278	148 ± 125	241 ± 54
		**1**	**1**	**1**	**3**	**4 ***	**7**	**2**	**2**	**1**	**1**

late	**Advax-CpG Control**	322 ± 405	134 ± 102	121 ± 180	32 ± 36	255 ± 639	ND	39 ± 14	ND	ND	ND
	**Advax-CpG Immune**	1132 ± 1145	253 ± 213	373 ± 408	83 ± 76	537 ± 758	36 ± 16	145 ± 101	41 ± 12	ND	ND
		4	2	3	3	2	x	4	x	x	x
	**Alum Control**	372 ± 389	181 ± 95	245 ± 236	56 ± 72	1492 ± 2949	102 ± 45	285 ± 297	56 ± 8	ND	ND
	**Alum Immune**	851 ± 884	267 ± 182	373 ± 290	233 ± 271	615 ± 467	79 ± 48	279 ± 512	38 ± 20	ND	ND
		2	1	2	4 *	−2	1	1	1	x	x

Table S3. Antigen-specific antibody titers from mice immunized with *Ov*-FUS-1 and either Advax-CpG, alum or AlT4 as the adjuvant. Serum was collected from immunized mice at the time of recovery following early and late recovery time points. IgG1 and IgG2ab specific for either *Ov*-103, *Ov*-RAL-2 or *Ov*-FUS-1 were measured in the serum using ELISA. Data shown are mean titers ± standard deviations. * p value ≤ 0.05 when compared to appropriate adjuvant-only controls at the at the same time point.

	**Anti-*Ov*-103**								
	**IgG1**								
	**Advax-CpG**			**Alum**			**AlT4**		
**Control**	0	±	0	0	±	0	0	±	0
**Immune Early**	63,352	±	70,244 *	764,012	±	270,577 *	1,054,163	±	466,021 *
**Immune Late**	65,094	±	61,280 *	746,343	±	226,494 *			
	**IgG2ab**								
	**Advax-CpG**			**Alum**			**AlT4**		
**Control**	0	±	0	0	±	0	0	±	0
**Immune Early**	5378	±	2856*	3018	±	1240 *	22,887	±	22,263 *
**Immune Late**	4460	±	5826*	3045	±	1895 *			
	**Anti-*Ov*-RAL-2**								
	**IgG1**								
	**Advax-CpG**			**Alum**			**AlT4**		
**Control**	0	±	0	0	±	0	0	±	0
**Immune Early**	273,559	±	244,832 *	2,146,736	±	450,007 *	3,230,477	±	4,199,062 *
**Immune Late**	66,364	±	83,142 *	1,342,119	±	388,793 *			
	**IgG2ab**								
	**Advax-CpG**			**Alum**			**AlT4**		
**Control**	0	±	0	0	±	0	0	±	0
**Immune Early**	33,256	±	23,829 *	13,288	±	10,318 *	21,962	±	20,970 *
**Immune Late**	16,845	±	13,479 *	9358	±	7725 *			
	**Anti-*Ov*-FUS-1**								
	**IgG1**								
	**Advax-CpG**			**Alum**			**AlT4**		
**Control**	0	±	0	0	±	0	0	±	0
**Immune Early**	425,142	±	316,201 *	3,596,149	±	780,522 *	4,630,715	±	2,070,432 *
**Immune Late**	126,603	±	98,256 *	2,612,567	±	570,184 *			
	**IgG2ab**								
	**Advax-CpG**			**Alum**			**AlT4**		
**Control**	0	±	0	0	±	0	0	±	0
**Immune Early**	48,203	±	34,627 *	22,881	±	12,085 *	65,635	±	46,502 *
**Immune Late**	27,609	±	17,860 *	15,634	±	7932 *			

Table S4. Antigen-specific antibody titers from NHPs immunized with *Ov*-FUS-1 and either Advax-CpG, alum or AlT4 as the adjuvant. Serum was collected from immunized NHPs at multiple time points throughout the duration of the experiment as indicated. Data shown are mean IgG titers ± standard deviations. * p value ≤ 0.05 when compared to controls at the at the same time point.

Days post-prime-immunization	Anti-*Ov*-103											
	PBS Control			Advax-CpG			Alum			AlT4		
13	18,527	±	158	316,650	±	477,308	226,465	±	3738	444,894	±	376,811
27	9298	±	9176	174,389	±	99,880	228,895	±	4606	**2,658,311**	**±**	**2,102,120 ***
43	13,001	±	7832	**1,545,434**	**±**	**1,740,825 ***	655,001	±	367,025	**1,749,459**	**±**	**1,482,943 ***
57	8775	±	8271	663,994	±	381,013	440,857	±	367,487	879,232	±	15,320
71	12,983	±	6788	**1,756,574**	**±**	**1,519,522 ***	663,577	±	380,493	**2,595,039**	**±**	**1,486,527 ***
85	4000	±	0	217,496	±	9415	208,891	±	9331	219,453	±	7048
98	4000	±	0	424,840	±	353,688	215,249	±	4786	627,131	±	355,038
210	4000	±	0	53,860	±	5379	40,803	±	21,655	53,705	±	6813

Days post-prime-immunization	Anti-*Ov*-RAL-2											
	PBS Control			Advax-CpG			Alum			AlT4		
13	32,765	±	27,222	442,484	±	381,308	167,708	±	93,083	335,637	±	479,695
27	30,545	±	24,199	226,186	±	5973	383,884	±	422,704	103,075	±	113,892
43	15,595	±	239	**10,153,475**	**±**	**5,883,752 ***	3,471,588	±	96,379	3,443,499	±	93,977
57	16,221	±	288	2,639,646	±	1,510,302	2,606,750	±	1,461,383	2,565,914	±	1,453,102
71	30,426	±	23,051	**13,986,030**	**±**	**203,418 ***	**6,886,217**	**±**	**5,819,676 ***	**6,897,297**	**±**	**5,774,291 ***
85	4000	±	0	857,032	±	13,250	638,741	±	357,607	225,878	±	1864
98	4000	±	0	844,876	±	976	222,306	±	6816	218,367	±	1029
210	7721	±	6445	428,350	±	366,134	107,073	±	84,755	57,488	±	1198

Table S5. Antigen-specific antibody titers compared between serum and diffusion chamber fluid from NHPs immunized with *Ov*-FUS-1 and either Advax-CpG, alum or Alt4 as the adjuvant. Antigen-specific IgG titers were measured by ELISA in the serum and diffusion chamber fluid at the time of diffusion chamber recovery (day 98 post-prime-immunization). Titers were measured in diffusion chambers without *O. volvulus* L3 (media only) or with *O. volvulus* L3 (media + L3). Total IgG specific for either *Ov*-103 or *Ov*-RAL-2 were measured in the serum using ELISA. Data shown are mean IgG titers ± standard deviations.

Sample type	***Ov*-103**								
	**Advax-CpG**			**Alum**			**AlT4**		
Serum	424,840	±	353,688	215,249	±	4786	627,131	±	355,038
Diffusion chamber - media only	106,870	±	62,142	103,180	±	67,069	67,375	±	50,060
Diffusion chamber - media + L3	94,422	±	58,367	70,505	±	57,786	55,469	±	21,257
**Sample type**	***Ov*-RAL-2**								
	**Advax-CpG**			**Alum**			**AlT4**		
Serum	222,306	±	6816	218,367	±	1029	844,876	±	976
Diffusion chamber - media only	688,209	±	363,412	275,427	±	165,271	313,433	±	591,216
Diffusion chamber - media + L3	492,139	±	153,198	265,527	±	193,707	144,050	±	69,331
